# Analysis on factors controlling organic matter enrichment in marine-terrestrial transitional shale of Member 1, Upper Carboniferous Keluke Formation, Huaitoutala area, east of northern margin of Qaidam Basin, China

**DOI:** 10.1371/journal.pone.0328397

**Published:** 2025-07-16

**Authors:** Tiangong Lai, Fei Xia, Guangnan Huang, Wenjin Liu, Da Sun, Fanming Meng, Weilong Wang

**Affiliations:** 1 National Key Laboratory of Uranium Resources Exploration-Mining and Nuclear Remote Sensing, Nanchang, Jiangxi, China; 2 School of Earth Sciences, East China University of Technology, Nanchang, Jiangxi, China; 3 China National Administration of Coal Geology, Beijing, China; Hefei University of Technology School of Resources and Environmental Engineering, CHINA

## Abstract

The organic-rich shale of the Member 1 of the Keluke Formation (in this paper referred to as Member 1) has been newly discovered as a good source rock reservoir in the western Delingha Depression, Huaitoutala area in the east part of the northern margin of Qaidam Basin, but the factors that control the enrichment of the organic matter in the shale are still unclear. In this paper, the paleo-climate, redox condition, paleo-salinity, hydrothermal activity and primary productivity of the dark shale in Member 1 are studied based on the analysis results of the total organic carbon (TOC), the element geochemistry and the whole rock mineral testing, and the enrichment mechanism of organic matter is discussed. Based on the vertical variation of the TOC contents, the Member 1 shale section is divided into two sub-sections (Sub-section I and Sub-section II), with the TOC content of Sub-section II (avg. 2.79%) being higher than the TOC content of Subsection I (avg. 0.92%). Similarly, the chemical index of alteration (CIA) values of Subsection II are also higher, indicating a hot, warm and humid paleoclimate, whereas the CIA values of Subsection I are low, indicating a rapidly cooling paleoclimate. The ratios of Sr/Ba and Rb/K_2_O are relatively high, indicating that the paleo-water body of Member 1 was mainly dominated by medium-high salinity water. The fluctuating EF_U_ and EF_Mo_ values and the low-moderate ratios of Th/U, V/Cr and C_org_/P indicate that the shale was formed in an oxic – dysoxic environment. The slightly higher ratios of Al/(Al + Fe + Mn), the slightly negative Eu anomaly, and the (Cu + Co + Ni)×10-Fe-Mn and Zn-Ni-Co ternary diagrams show that the shale was affected by atypical (weak) hydrothermal activity with relatively high terrigenous clastic input. The P/Al, Cu/Al and Zn/Al ratios show that the primary productivity of the surface water was relatively high with an upward gradually increasing trend. The TOC is significantly correlated with the indicators of palaeo-climate, palaeo-salinity, terrigenous clastic input and primary productivity, indicating that the enrichment of organic matter in the shale of Member 1 is mainly controlled by the high primary productivity, the brackish – salt water environment and the dysoxic preservation condition in a warm and humid climate. In addition, the terrigenous clastic input (adsorption of clay minerals) also plays important roles. Based on these results, a two-section organic matter enrichment model of this Member is established.

## 1. Introduction

China has made great progress in shale gas development in recent years, with the geological reserves up to 80 × 10^12^ m^3^ being proved and 3 types of shale sedimentary environment including marine facies, marine-terrestrial transitional facies and terrestrial facies being determined [1–4]. Among which, the marine shale gas has been efficiently developed, and the production breakthrough of 1 × 10^12^m^3^ has been achieved [1]. However, research on the transitional shale gas still remains in the initial stage, and studies on controlling factors and the enrichment model of the organic matter in the transitional shale are still weak, although this type of shale gas has abundant resources and huge development potentials. The Qaidam Basin, located in the Qinghai Province on the northern margin of the Qinghai-Tibet Plateau, is an important Mesosoic and Cenozoic large continental sedimentary basin in western China, with extremely rich mineral resources [5–7]. It is also one of the important oil-bearing basins in northwest China, with the Mesosoic and Cenozoic strata as the main oil and gas bearing horizons [8]. Relevant studies have shown that the Paleocene-Lower Triassic strata in this basin belong to a marine or marine-terrestrial transitional sedimentary system, in which the Carboniferous residual strata are widely distributed, with relatively limited damage degree from metamorphism and large oil and gas potential, and have been expected to be new oil- and gas- bearing horizons for “increasing reserves and production” of the basin [9–13]. Up to now, wells including CY2, QDD1, and QDC1 have been drilled in and around the Onan Depression in the east part of the northern margin of the Qaidam Basin (NMQB), and some new oil and gas discoveries and hydrocarbon potentials have been obtained in the Upper Carboniferous Keluke Formation (C_2_*k*) [11], which makes this formation a focus for oil and gas investigation and exploration in the basin. Compared with the organic matter enrichment mechanism of the shale of the Longmaxi Formation and Wufeng Formation in Sichuan Basin, which has been deeply studied with the key factors controlling organic matter enrichment being determined as the warm and humid paleoclimate and salinity stratification [14,15], the study on the Keluke Formation of the NMQB is still relatively weak. Although the organic matter composition of the Keluke Formation source rocks has been systematically analyzed, and the contributions of bio-silicon and marine lower organisms to hydrocarbon generation have been pointed out by Shi et al. (2023) [13], the differences of the main factors controlling the organic matter in different intervals, especially the synergistic effect between the terrestrial clastic input and the paleoenvironment, have not been determined.

The factor controlling organic matter enrichment is a key issue in the formation of organic-enriched shale series. Detailed studies on the enrichment of the organic matter in the shale series in marine and transitional environment have been extensively conducted, but the enrichment mechanism has not been fully understood due to the complex physical and chemical processes [16–23]. Currently, there exist 2 main types of models for the enrichment of organic matter – the productivity model and the preservation model. The productivity model emphasizes that the primary productivity of surface water is the key factor controlling organic matter enrichment [24], which relates to the organic carbon fluxes that may be influenced by the paleoclimate, the clastic input and the hydrothermal fluids [25–30]. In contrast, the preservation model suggests that the redox condition, paleo-salinity, the sedimentation rate and sea level change act as the key factors in organic matter enrichment, especially the redox of the benthic water body [31–35]. In summary, the high productivity of surface water provides the material basis for organic matter enrichment, and the preservation condition and the sedimentation rate are the key factors affecting the preservation of organic matter.

At present, the research on shale gas in the western Delingha Depresion in the eastern NMQB is still a blank. In this study, the thick, dark shale have been discovered for the first time in the Member 1 of the Keluke Formation in Huaitoutala area through feild geological surveys. In order to explore the organic matter enrichment mechanism, the Olong-Buluke Section is taken as the research object, and the analysis of TOC content, element geochemistry, and whole rock minerals is carried out. By combining with the indicators of paleoclimate, paleo-salinity, redox condition, hydrothermal activity and primary productivity, the main factors that control the organic matter enrichment in the shale of Member 1 of the Keluke Formation are discussed, and an organic matter enrichment model is established, providing a new theoretical basis for shale gas exploration in the eastern region of the NMQB.

## 2. Regional geological setting

**Fig 1:**
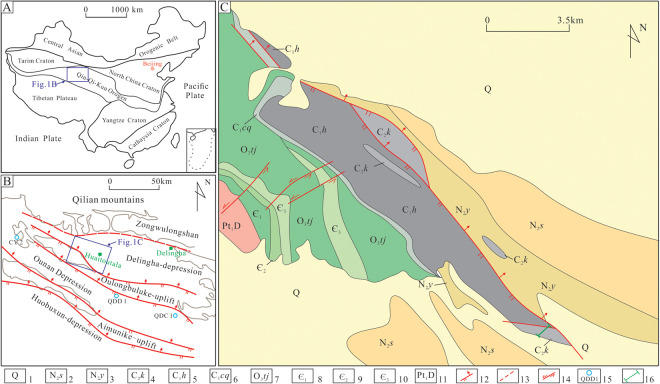
(A) Geotectonic location of the Qaidam Basin. (B) Structure outline of the eastern region of the NMQB. (C) Geological sketch of Huaitoutala area. 1-Quaternary system; 2-Pliocene Shizigou Formation; 3-Pliocene Youshashan Formation; 4-Upper Carboniferous Keluke Formation; 5-Lower Carboniferous Huaitoutala Formation; 6- Lower Carboniferous Chengqianggou Formation; 7-Upper Ordovician Tanjianshan Group; 8- Lower Cambrian Series; 9- Middle Cambrian Series; 10-Upper Cambrian Series; 11-Paleoproterozoic Dakendaban Group; 12-Reverse fault; 13-Inferred fault; 14-Strike-slip fault; 15- Oil and gas drilling well; 16- Location of the section in this study.

The Qaidam Basin lies in the eastern part of northwestern China, developed tectonically within the Qing (Qingling)-Qi(Qilianshan)-Kun (Kunming) orogenic belt, bordering the Tali Plate to the north and the Qinghai-Tibet Plateau to the south, and echoing the Yangtze Plate to the southeast (Fig 1A). The Nanqilian-zongwulong tectonic belt, the Delingha Depression, the Olong-Buluke bulge, the Onan Depression, and the Emnik bulge develop in the eastern region of the NMQB from north to south in turn (Fig 1B), forming a tectonic pattern of “ 3 bulges mixing with 2 depressions”. The Upper Carboniferous Keluke Formation (C_2_*k*) corresponds to the Benxi Formation (C_2_*d*) in northern China, belonging to the Bashikirian-Moscovian period (about 323.2–307.1 Ma) [15]. The results of the tectonic setting studies show that the Kunnanyang Block constantly subducted northward in the Carboniferous period, with the tectonic mechanism dominated by the Cambian-Devonian compression setting shifting to the extensional setting, and thus placed the eastern region of the NMQB generally in an arc-back setting and made the Olong-Buluke micro-blocks (Onan Depression, Olong-Buluke Bulge, and Delingha Depression) subside [36–39]. During the late Early Carboniferous to Early Permian, the eastern region of the NMQB was mainly influenced by the north-to-south transgression and superposition of the Zongwulong Trough, and the clastic rock and carbonate mixed sediments from the lagoon – barrier island – offshore shelf sedimentary system developed, which belongs to a typical marine-terrestrial interactive sedimentary facies [40]. The thick shale deposited around the lagoon facies, and formed good hydrocarbon source rock reservoir.

**Fig 2:**
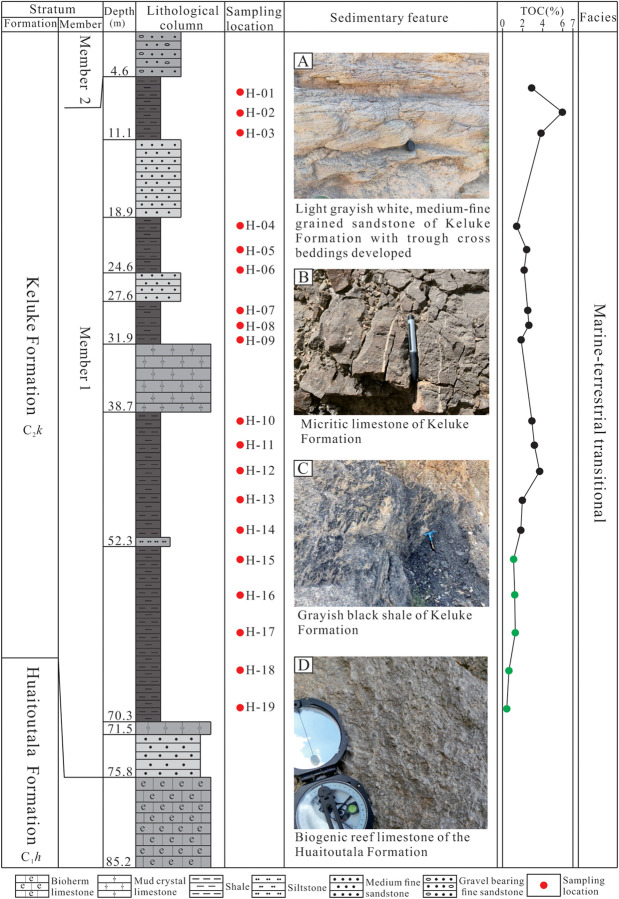
Lithologic column, sampling position, sedimentary characteristics and vertical change trend of TOC content of Member 1 of Keluke Formation along the Olong-Buluke Section.

The Olong-Buluke Section lies in Huaitoutala area in the eastern region of the NMQB (Fig 1C), with the exposed strata along this section mainly including the Lower Carboniferous Huaitoutala Formation (C_1_*h*) and the Upper Carboniferous Keluke Formation (C_2_*k*) (Fig 2). The Huaitoutala Formation is mainly composed of thick, dark-gray reef limestones (Fig 2D), rich in a variety of biological fossils such as brachiopod, coral, and crinoid stem fossils. In contrast, the Keluke Formation is mainly composed of thick, dark shale (Fig 2C), mostly intercalated with medium-thick, layered micritic limestone (Fig 2B), medium-fine sandstone and coarse-grained sandstone, with trough cross beddings developed (Fig 2A). Based on the lithological characteristics, the Keluke Formation is divided into the 2 members – Member 1 and Member 2 (Fig 2).

## 3. Sample collection and analytical method

In this study, 19 shale samples collected from Member 1 along the Olong-Buluke Section are selected and labelled from H-01 to H-19 according to sampling order from top to bottom, as shown in Fig 2. The experimental analysis of the shale samples can provide important data for studying the organic matter enrichment mechanisms in shale of this Member. The items analyzed include the total organic carbon (TOC) content, the major and trace element content, and the XRF quantification of whole rock minerals. All these analyses are carried out by the State Key Laboratory of Nuclear Resources and Environment, East China University of Science and Technology. The samples collected are ultrasonically washed with deionized water and dried, and then crushed to 200mesh by a Lechi Tungsten Carbide Disc Grinder (RS200) for use.

The TOC content determination is conducted by using the determination method of the total organic carbon in sedimentary rocks, and samples are analyzed by means of the CS-230 Carbon Sulfur Analyzer following the GB/T19145-2003. The samples are first soaked in 5% dilute hydrochloric acid, and kept in a water bath pot at 80°C for more than 2 hours until the carbonate rocks are completely removed; then they are washed until neutral and dried, and analyzed by means of the CS-230 Carbon-sulfur Analyzer, with an analysis accuracy >0.1%. The results are shown in S1 Table.

The XRD quantitative analysis of the whole rock minerals is conducted following the national standard (SY/T5163-2018). First, 5g shale sample is weighed and placed in the groove of the XRD sample stage, and then the surface of the groove is compacted tightly by using smooth flat glass to ensure that the surface of the sample is flat. The Rigaku SmartLab9 X-ray Diffractometer is used for sample analysis with the following test conditions: Cu-Kα radiation; working voltage and current being 40kV and 40mA, respectively; the divergent slit and the scattering slit being both 1°, and the receiving slit being 5.5 mm; the continuous scanning mode being adopted, with the scanning range of 5° to 80° (2θ), the scanning time of 29.845s, and the scanning step size of 0.015°. The analysis accuracy is > 3%. The results are also shown in S1 Table.

The major element analysis is conducted through the fusion casting glass disk method used in the X-ray fluorescence spectrometry chemical analysis of the refractory materials following the GB/T21114-2007. The 0.5 - 1g shale sample is weighed and placed in a clean ceramic crucible, and the crucible, the sample and the crucible+sample are weighed respectively and the results are recorded for determining the loss of the sample in the subsequent calcination process. The crucible is placed in a muffle furnace and kept calcining at 920°C for 3–4 hours to remove organic matter, and then taken out and quickly placed in a desiccator to cool. Afterwards, the sample+crucible and the crucible alone are weighed at room temperature. The calcined powder sample of 0.5g is weighed and added with 8 times the sample weight of Li_2_B_4_O_7_, and then mixed evenly. The mixed sample is placed into a special platinum crucible, added 4 drops of NH_4_Br, and then put in the TR-1000S automatic sample melting furnace at 1100°C for about 10 minutes (static melting for 3 minutes, no-rotation swinging for 2 minutes, and swing and rotating for 5 minutes) to prepare uniform glass slices. The Rigaku 100e Wavelength Dispersive X-ray Fluorescence Spectrometer is used for testing, with the analysis accuracy >2%. The results are shown in S2 Table.

The trace element analysis is conducted by using the silicate rock chemical analysis method following the GB/T14506.30-2010. The 40g sample is weighed and placed in a clean and dry TeFlon melting tank. The sample is added with 0.5mL (1 + 1)HNO_3_ and 1mL HF solution, and then covered and ultrasonically oscillated for 10–15 minutes, and put on a 150°C electric heating plate until nearly dry. Again, the sample is added with 0.5mL (1 + 1)HNO_3_ and 1.5mL HF solution, covered, and inserted into the heat shrink tube and placed into the stainless steel vessel, which is then placed into the oven, heated with the temperature gradually increasing to 200°C, and kept at this temperature for 5 days. The sample vessel is taken out afterwards, uncovered, and steamed until nearly dry. The sample is added with 2mL (1 + 1)HNO_3_ solution, covered, inserted into the heat shrink tube and placed into the stainless steel vessel, and then placed into the 150°C oven and kept for 5 hours. Repeat this process twice to ensure complete dissolution of the sample. The sample solution is then transferred to a PE bottle, added with 1mL 500ppb Indium (In) standard solution, and diluted to 50mL with 1% HNO3 solution. The test is carried out by using the PE Elan6000 Inductively Coupled Plasma-Mass Spectrometer (ICP-MS) with the analysis accuracy >5%. The results are shown in S3 Table.

## 4. Analysis and test result

### 4.1. TOC content and mineral composition

**Fig 3:**
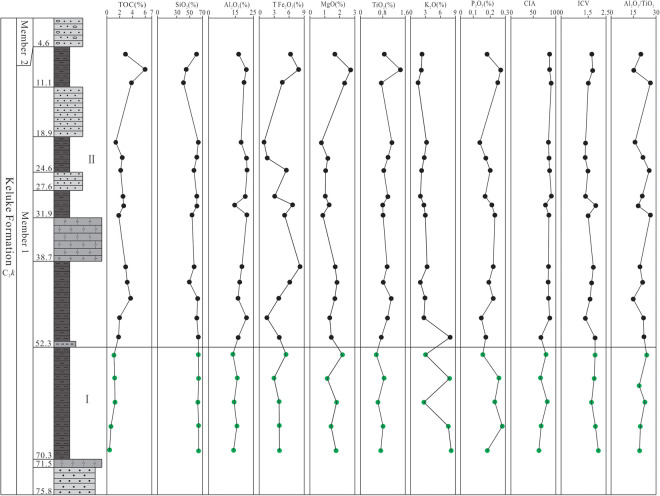
Vertical variation of the values of the main elements and related parameters in Member 1 of Keluke Formation.

The TOC content data of the shale samples from Member 1 in the study area are listed in S1 Table and shown in Fig 2. As can be seen from Fig 2, the TOC values vary significantly in the vertical direction, ranging from 0.43% to 5.96% (avg. 2.38%, n = 19; S1 Table). Based on the vertical distribution characteristics of the TOC values, the shale section of Member 1 is divided into 2 sub-sections (Fig 3). Sub-section I (located in the lower part of the shale section, with the depth of 52.3–70.3 m) has the lower TOC contents, ranging from 0.43% to 1.26% (avg. 0.90%, n = 5), with the changing trend of the TOC values increasing gradually upward; Sub-section II (located in the middle and upper part of the shale section, with the depth of 4.6–52.3 m) has the higher TOC contents, showing the fluctuation characteristics in the TOC value variation, with the values ranging from 1.38% to 5.96% (avg. 2.96%, n = 14), obviously higher than the values in Sub-section I (S1 Table).

The whole-rock mineral compositions of 19 shale samples are shown in S1 Table. XRF analysis results indicate that clay (accounting for 32.0–53.5%, avg. 41.59%, n = 19) is the most abundant mineral component in the shale samples, followed by quartz (24.70–50.50%, avg. 40.88%, n = 19), potassium feldspar (1.30–12.70%, avg. 3.71%, n = 19), plagioclase (1.70–7.90%, avg. 3.09%, n = 15), calcite (1.50–5.30%, avg. 2.78%, n = 8), ankerite (3.20–10.70%, avg. 6.69%, n = 14), pyrite (2.00–5.40%, avg. 3.11%, n = 11), siderite (1.60–5.90%, avg. 2.84%, n = 19), gypsum (0.20–2.50%, avg. 1.28%, n = 9), and halite (0.80%, n = 1) (S1 Table). The TOC content in the samples shows a significantly positive correlation with clay mineral content (R = 0.557, n = 19), and an obviously negative correlation with the content of quartz (R = −477, n = 19), potassium feldspar (R = −0.463, n = 19) and ankerite (R = −0.576, n = 14) (S4 Table).

### 4.2. Element geochemistry

#### 4.2.1. Major element.

**Fig 4:**
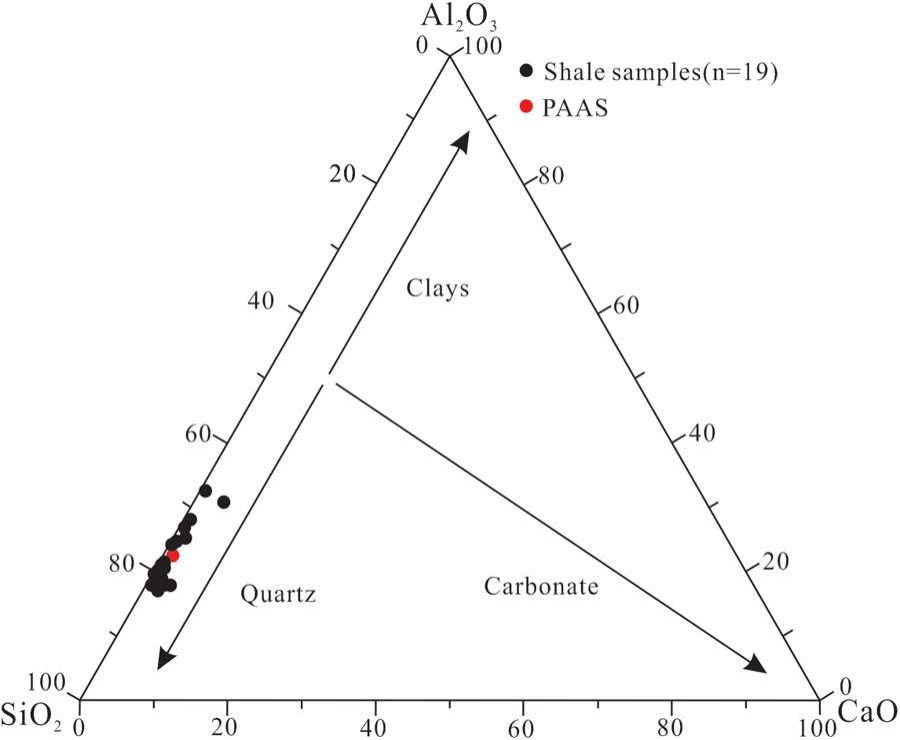
Ternary diagram showing the relative content of SiO_2_, CaO, and Al_2_O_3_ in shale samples from Member 1 of Keluke Formation. Data of PAAS are quoted from Taylor and McLennan (1985) [42].

The XRF analysis results show that SiO_2_ (41.41–64.06%, (avg. 58.68%, n = 19) and Al_2_O_3_ (13.59–21.44%, (avg. 17.82%, n = 19) are the most important oxides in the shale samples from Member 1, followed by Fe_2_O_3_T (0.97–8.19%, (avg. 4.53%, n = 19), K_2_O (1.51–8.15%, (avg. 3.67%, n = 19) and MgO (0.77–2.73%, (avg. 1.54%, n = 19). In contrast, CaO (0.05–2.87%, (avg. 0.74%, n = 19), Na_2_O (0.11–0.87%, (avg. 0.40%, n = 19), TiO_2_ (0.57–1.43%, (avg. 0.89%, n = 19), P_2_O_5_ (0.12–0.37%, (avg. 0.21%), and MnO (0.01–0.38%, (avg. 0.09%, n = 19) all have average content values less than 1.0% (S2 Table), with the vertical content variation shown in Fig 3. In the ternary diagram proposed by Ross and Bustin (2009) [41] in which the shale is regarded as a mixture of 3 end-member oxides, i.e., SiO_2_, Al_2_O_3_ and CaO, all samples fall into the end-element region close to SiO_2_ (Fig 4), indicating that shale in Member 1 has higher SiO_2_ content than Al_2_O_3_ and CaO, and is characterized by high siliceous content.

The content of SiO_2_ is positively correlated with the content of K_2_O (R = 0.475, n = 19) and quartz (R = 0.726, n = 19) (S4 Table), indicating that silicon mainly exists in felsic minerals such as quartz and potassium feldspar. The Al_2_O_3_ content is positively correlated with the clay mineral content (R = 0.784, n = 19; S4 Table), indicating that Al exists mainly in clay minerals. The MgO content is negatively correlated with Fe_2_O_3_T (R = −0.545, n = 19), CaO (R = −0.581, n = 19) and calcite (R = −0.864, n = 19) (S4 Table), indicating that Mg exists mainly in ferruginous and carbonate minerals. The TiO_2_ content is positively correlated with Al_2_O_3_ (R = 0.551, n = 19) and clay mineral content (R = 0.542, n = 19) (S4 Table), indicating that Ti is associated with clay minerals.

#### 4.2.2. Trace element.

The analysis data of trace elements in the shale samples of Member 1 are listed in S3 Table. V (72.49–197.43 ppm, avg. 129.12 ppm), Rb (72.12–228.24 ppm, avg. 137.38 ppm), Sr (58.29–564.17 ppm, avg. 159.39 ppm), Ba (132.41–729.47 ppm, avg. 366.63 ppm), Cu (59.41–188.79 ppm, avg. 103.90 ppm), Zn (73.56–178.78 ppm, avg. 115.84 ppm) and Zr (99.29–331.97 ppm, avg. 219.67 ppm) are the highest abundant elements in the shale samples studied (each having the average value higher than 100 ppm). These high content elements are followed by Cr (38.52–95.73 ppm, avg. 71.32 ppm), Ni (8.82–66.57 ppm, avg. 28.19 ppm), Ga (15.94–33.01 ppm, avg. 23.43 ppm) and Y (19.17–41.92 ppm, avg. 30.64) (S3 Table). In contrast, Sc (7.88–20.95 ppm, avg. 14.60 ppm), Co (3.19–24.43 ppm, avg. 12.73 ppm), Mo (0.16–1.78 ppm, avg. 0.61 ppm), Cs (8.08–18.42 ppm, avg. 11.05 ppm), Th (10.03–19.41 ppm, avg. 14.53 ppm), U (1.72–4.20 ppm, avg. 2.96 ppm), Nb (8.71–21.65 ppm, avg. 16.59 ppm), Ta (0.46–1.72 ppm, avg. 1.09 ppm) and Hf (2.81–7.62 ppm, avg. 5.51 ppm) have the average content values less than 20 ppm (S3 Table). The content of Co, Ni, Sr, or Hf is significantly positively correlated with Fe_2_O_3_ and MgO (S4 Table), implying that these elements mainly come from mafic minerals.

**Fig 5:**
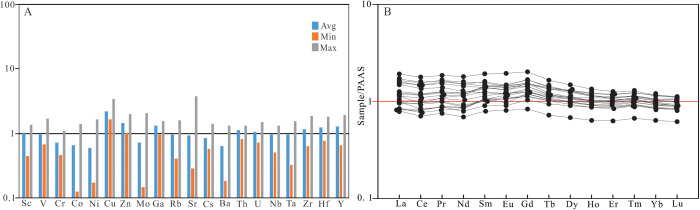
Enrichment factors (EF) of trace elements (A) and PAAS normalized REE distribution (B) of shale samples from Member 1 of Keluke Formation.

The trace element enrichment factor (EF) has been widely used to evaluate the enrichment or depletion of the trace elements with the calculation formula EF_X_=(X/Al) _sample_/(X/Al) _PAAS_ [43], where X and PAAS represent the certain element studied and the post-Archean Australian shale, respectively [42]. The value EF_X _< 1 and EF_X _> 1 are used to represent the depletion and enrichment degree, respectively. The EF values are shown in S5 Table and Fig 5A. It can be seen that the trace elements of Cu (1.659–3.39, avg. 2.18), Zn (1.03–1.97, avg. 1.44), Sc (0.44–1.36, avg. 1.02), Ga (0.95–1.54, avg. 1.30), Th (0.82–1.31, avg. 1.12), U (0.72–1.50, avg. 1.06), Zr (0.64–1.87, avg. 1.17), Hf (0.76–1.80, avg. 1.23), and Y (0.65–1.95, avg. 1.28) (S5 Table) are weakly rich in the shale samples studied (Fig 5A); the trace elements of V (0.67–1.71, avg. 0.97), Cr (0.46–1.07, avg. 0.73), Co (0.12–1.38, avg. 0.65), Ni (0.17–1.63, avg. 0.59), Mo (0.15–2.04, avg. 0.73), Rb (0.40–1.59, avg. 0.97), Sr (0.28–3.79, avg. 0.94), Cs (0.58–1.40, avg. 0.83), Ba (0.18–1.33, avg. 0.64), Nb (0.51–1.31, avg. 0.97) and Ta (0.32–1.55, avg. 0.95) (S5 Table) all show the characteristics of slight depletion (Fig 5A). The PAAS normalized distribution pattern of the elements in the shale shows that all sample curves tend to be horizontal (Fig 5B), where Eu (δEu) and Ce (δCe) values have weak negative anomalies (S3 Table), indicating that the shale originates from the upper crust, similar to the source of the post-Archean Australian shale.

## 5. Discussion

### 5.1. Terrigenous clastic input and paleoclimate reconstruction

**Fig 6:**
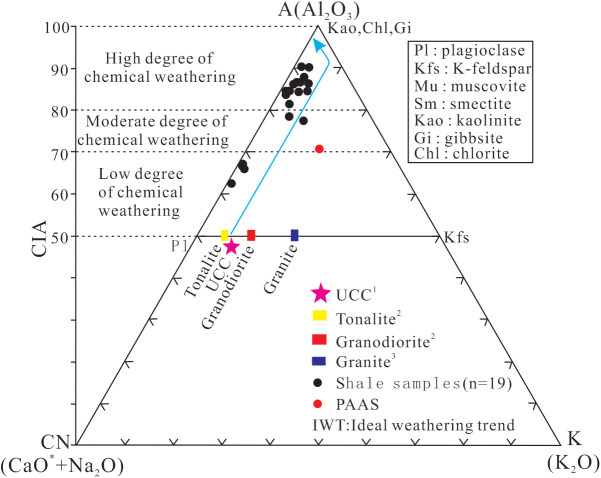
Chemical composition of the shale samples from Member 1 of Keluke Formation, the A-CN-K ternary diagram, and the CIA variation. ^1^Taylor and Mclennan(1985) [42]; ^2^Best(2011) [57]; ^3^Condie(1993) [58].

**Fig 7:**
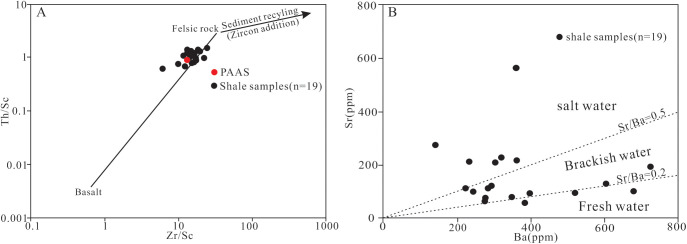
(A) Cross plot of Zr/Sc and Th/Sc ratios of the shale samples from Member 1 of Keluke Formation. (B) Schematic diagram showing the salinity of ancient water.

The chemical weathering of rocks is closely related to paleoclimate change [44,45]. Typically, alkali and alkali earth metals (K, Na, Ca, and Mg) are the first ones being leached out; In contrast, Al and Ti remain in the weathering source area [44]. In this paper, the chemical index of alteration (CIA) is used to evaluate the intensity of surface chemical weathering and the paleoclimate environment. The CIA values generally increase with the intensity of chemical weathering, indicate a gradually warmer climate [46–48]. The calculation formula for CIA is CIA=[Al_2_O_3_/(Al_2_O_3_+CaO* + Na_2_O+K_2_O)]×100, where CaO* represents the content of CaO only in silicate minerals. Therefore, it is necessary to remove the CaO content in non-silicate minerals when calculating CIA, and this is achieved by the indirect calculation method of CaO* = CaO-P_2_O_5 _× 10/3 proposed by Mclennan et al. (1993) [49]. The previous studies show that the CIA values between 50 and 70 indicate that the paleoclimate of weathering period was cold and arid, and the chemical weathering degree was weak; the CIA values between 70 and 80 indicate that the paleoclimate was warm and humid, and the chemical weathering degree was moderate; and the CIA values >80 indicate a hot and humid paleoclimate with a strong degree of chemical weathering [50]. Although potassium metasomatism may decrease the CIA calculation value, it can be distinguished by using the A (Al_2_O_3_) -CN (CaO* + Na_2_O) -K (K_2_O) ternary diagram [47,48,51,52]. In Fig 6, the samples not influenced by potassium metamorphism are clustered near the line parallel to the ideal weathering trend line (A-CN), otherwise the samples will deviate from the A-CN trend line [47,48,52]. The shale samples from Member 1 in this study are mostly clustered along the line parallel to A-CN axis in the A-CN-K diagram, showing that these shale samples have not undergone potassium metasomatism during the diagenetic process (Fig 6), and thus CIA can be reliaby used in this study. Besides, the material transport distance can also affect CIA value; the longer the transport distance is, the higher the CIA values are [47]. On the other side, the ratio of SiO_2_/Al_2_O_3_ can reflect the compositional maturity and sedimentary recirculation of the sediments. The ratio value less than 10 indicates the lower degree of sedimentation recirculation and compositional maturity, and thus the shorter transport distance [53,54]. The SiO_2_/Al_2_O_3_ ratios of the samples studied range from 2.04 to 4.68 (avg. 3.40; S2 Table), significantly lower than 10, indicating the low degree of sedimentation recirculation and compositional maturity of the shale. In addition, the sedimentation recirculation can also lead to the enrichment of the heavy minerals [49]. Therefore, the ratios of Zr/Sc and Th/Sc can be used to effectively evaluate the compositional change and sedimentation recirculation in the source area [49,55,56]. The Th/Sc ratios are low, ranging from 0.65 to 1.70 (avg. 0.26; S3 Table); while the Zr/Sc ratios are relatively high, ranging from 6.12 to 24.59 (avg. 15.62; S3 Table). In Fig 7A, all samples plot fall into the region below the sedimentation circulation line and show an obvious linear relationship, indicating the lower degree of sedimentation recirculation and shorter transport distance. In conclusion, the CIA values can truthfully reflect the chemical weathering condition and the corresponding paleoclimate characteristics of the shale samples from Member 1.

Overall, the CIA values of the shale samples from Member 1 in the study area range from 62.73 to 90.43, with an average of 80.87, generally increasing at the beginning and then tending to be stable from bottom to top (Fig 3), indicating that the paleoclimate has experienced a variation trend from cold and arid to warm, hot and humid periods. The CIA values of the shale from Sub-section I are 62.73–81.39 (avg. 71.25, n = 5), indicating that the paleoclimate was dominantly cold and arid, providing weak chemical weathering conditions; the CIA values of Sub-section II range from 66.91 to 90.43 (avg. 84.30, n = 14), indicating that the paleoclimate was basically dominated by warm, hot and humid conditions, with moderate to strong chemical weathering.

### 5.2. Palaeoenvironment

#### 5.2.1. Paleosalinity.

The salinity of ancient water body is an important indicator for distinguishing sedimentary environments (marine, marine-continental transitional and continental facies). The solubility of Sr in seawater is relatively high, while Ba is prone to accumulate in freshwater environments. Although Rb is similar in geochemical behavior to K, it is relatively more enriched in seawater. Therefore, the ratios of Sr/Ba and Rb/K_2_O can be used as effective indicators to determine the paleo-salinity of sedimentary water, with content of Rb being multiplied by 1000 when calculating the Rb/K_2_O ratio [59,60]. However, the influence of diagenesis needs to consider when applying the ratios of Sr/Ba and Rb/K_2_O ratios in determining the paleo-salinity. Sr may be reactivated during the carbonate recrystallization process [61]. The calcite content of the shale samples from Member 1 of the Keluke Formation in the study area are low (avg. 2.78%), indicating that the effect of Sr reactivation is limited. Furthermore, Rb can by stably adsorbed by clay minerals [42], and thus the Rb/K_2_O ratio is less disturbed by diagenesis. Generally, the Sr/Ba ratios >0.5, 0.2 ≤ Sr/Ba ≤ 0.5 and <0.2 indicate a salt water, a brackish water and a fresh water environment, respectively [8,62–64], and Rb/K_2_O ratios >6, 4–6 and <4 indicate a salt water, a brackish water and a fresh water deposition environment, respectively [65].

**Fig 8:**
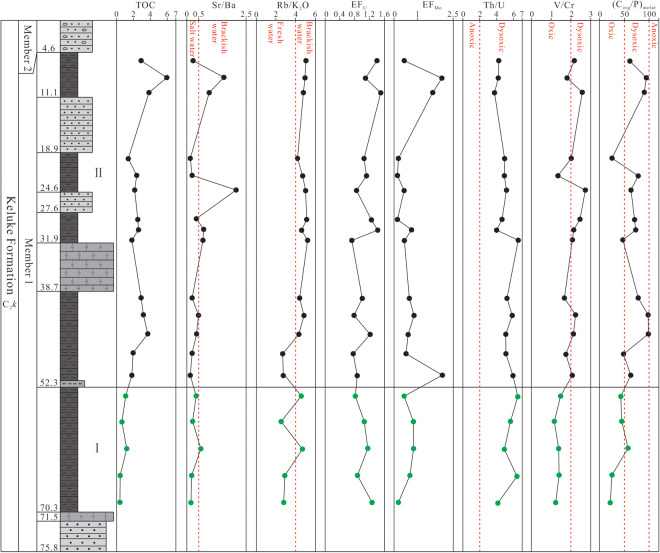
Vertical variation trend of the TOC, the paleosalinity indicators and redox indexes of the shale samples from Member 1 of Keluke Formation.

The shale samples from Member 1 in this study mostly fall into the brackish and salt water environment regions in the Ba-Sr discrimination diagram (Fig 7B), and the Sr/Ba ratios range from 0.15 to 2.08 (avg. 0.54; S3 Table), also indicating the brackish-salt water environment (S3 Table). Therefore, it can be inferred that the sedimentation period of Member 1 is basically dominated by brackish-salt water environment. As can been seen from the Sr/Ba value trend chart (Fig 8), the Sr/Ba values of Sub-section I showed a trend of increasing at the beginning and then decreasing from bottom to top, indicating regressive and transgressive events in the basin during the sedimentation period. The Sr/Ba values of Sub-section II remain steadily at about 0.3 in the the initial stage, and fluctuate frequently in the later stage, indicating that frequent regressive and transgressive events occurred in the sedimentary basin in this period.

The Rb/K_2_O ratios in this study range from 2.54 to 5.21 with an average of 4.18, indicating the brackish-salt water environment in general, consistent with the paleo-environment property inferred from the Sr/Ba ratios. The Rb/K_2_O values of Sub-section I range from 2.54 to 4.68 (avg. 3.50), showing the changeable fresh water to brackish water environment, whereas the Rb/K_2_O ratios of Sub-section II range from 4 to 6, basically indicating the brackish water environment (S2 Table, Fig 8). The comparison studies between the Sr/Ba and Rb/K_2_O ratios find that there are obvious synchronous fluctuation in the values, indicating that the water paleosalinity has obvious cyclicity during sedimentation. In addition, the correlation between CIA value, Sr/Ba ratio, and Rb/K_2_O ratio is also discussed, with no obvious correlation found between Sr/Ba ratio and CIA value (R = 0.403, n = 19; S4 Table), and a significantly positive correlation found between the Rb/K_2_O ratio and the CIA value (R = 0.784, n = 19; S4 Table). These results indicate that the paleosalinity of the water body had little relationship with the paleoclimate change during sedimentation period, and further verify that the change of the water salinity during the sedimentation period was closely related to the regressive and transgression.

#### 5.2.2. Redox condition.

Anoxic condition is one of the key factors for the enrichment and preservation of organic matter, and some sensitive elements such as Cr, Mo, U, Th, V, etc. have been normally used in identifying the redox condition of ancient water body [66–68]. As redox sensitive elements, Th and U have different geochemical behaviors, with Th being relatively stable in sedimentary process, while U being prone to enrichment under reducing conditions, thus the ratio of the two elements can effectively reflect the changes in redox conditions [67]. The valence states of V and Cr change significantly under different redox conditions, with V being prone to enrichment under reducing conditions, while Cr existing stably under oxidizing conditions [43]. Therefore, the Th/U and V/Cr ratios can be used as indicators for redox condition reconstruction. In contrast, the Ni/Co ratio is susceptible to the input of land-based clastics and has a lower resolution degree for dysoxic environments [43]; the Mo/TOC ratio is more applicable to long-term stable marine anoxic basins [69].

**Fig 9:**
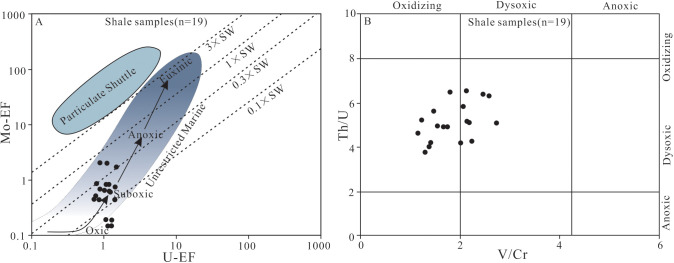
(A) Distribution of EF_U_ and EF_Mo_ (A) and cross plot of Th/U and V/Cr (B) of shale samples from Member 1 of Keluke Formation. The dashed lines indicate the different times of the corrsponding value in seawater in mole ratio (0.1 times, 0.3 times, 1times, and 3 times, respectively).

The U and Mo content of the shale samples from Member 1 shows no correlation with the content of Al_2_O_3_ (R = 0.208 and 0.092, n = 19; S2 Table), similiar to the research results of the Sohnari Member, Laki Formation in the Indus Basin in southern Pakistan [70]. The study also found that the enrichment of U-Mo in the marine-terrestrial transitional environment is controlled by redox conditions rather than terrigenous input. The U_EF_ and Mo_EF_ values of the shale samples from Member 1 fluctuate significantly, ranging from 0.72 to 1.50 (avg. 1.06) and 0.15 to 2.04 (avg. 0.73), respectively (S5 Table), with the enrichment degree being lower than that in a typical anoxic marine environment (Mo_EF_ > 5), which may be related to the salinity changes in marine-terrestrial transitional facies. The weak enrichment or depletion of U and Mo indicates that the shale of Member 1 was formed in an oxic – dysoxic environment (Fig 8). In addition, the EF_U_-EF_Mo_ covariant diagram can also be used to distinguish the redox condition of sedimentary water body [43,69]. In the EF_U_-EF_Mo_ cross plot (Fig 9A), most of the shale samples have EF_Mo_/EF_U_ ratios 0.3 times those of the modern seawater, with a few samples having EF_Mo_/EF_U_ ratios 0.1 or 1.0 times those of the modern seawater [69]. These indicate that the water body was mainly in an dysoxic-anoxic environment in the sedimentation of the shale in Member 1.

The Th/U value increases with the water oxidation degree [71], whereas the V/Cr value decreases with the water oxidation degree [72]. The Th/U ratios between 0 and 2 represent anoxic environment; between 2 and 8 represent dysoxic environment; and greater than 8 represent oxic environment [71,73]. The Th content is significantly positively correlated with U (R = 0.699, n = 19); S4 Table), indicating that the Th/U ratio can effectively reflect redox condition. The Th/U ratios range from 3.75 to 6.54 (avg. 5.13) (S3 Table, Fig 9), greater than the standard threshold in marine anoxic environment (0–2), but lower than that in oxidizing environment (>8), showing obvious transitional characteristics, indicating that the shale in Member 1 of the Keluke Formation was formed in a dysoxic water environment. Generally, the V/Cr ratios less than 2 indicate an oxic environment; between 2 and 4.25 indicate an dysoxic environment; and greater than 4.25 indicate an anoxic environment [72]. The V/Cr ratios of the shale samples range from 1.16 to 2.73 (avg. 1.83) (S3 Table, Fig 8), near the lower limit of the ratio range (2–4.25) in the marine dysoxic environment, indicating that the shale was formed in an oxic – dysoxic water environment. In the cross plot of Th/U and V/Cr, all the studied samples fall into the oxic and the dysoxic regions (Fig 9B), also indicating that Member 1 was formed in the oxic-dysoxic water environment.

The ratio of organic carbon to phosphorus content (C_org_/P) in sediments correlates organic carbon with phosphorus occurrence in the sediments, and can act as a reliable indicator for evaluating the redox condition of the bottom water in sedimentary environment [74–76].The difference in the anoxia degree of water controls phosphorus cycle between sedimentary water and sediments, causing corresponding change in C_org_/P ratio, which can be used to predict the redox condition of sedimentary environment. Previous studied have shown that the ratios of C_org_/P < 50, 50–100 and >100 represent oxic, dysoxic and anoxic environments, respectively [74,75]. In this study, the C_org_/P ratios are 20.92–99.00 (avg. 62.16; S2 Table, Fig 8), with most of which being 50–100, and a small amount being less than 50, again indicating that Member 1 was formed in the oxic-dysoxic water environment.

In summary, based on the study results of the indicators including V, U and Mo, Th/U, V/Cr, EF_U_ and EF_Mo_ of the dark shale samples from Member 1 in Huaitoutala area, it is concluded that the water in the sidimentary period of Member 1 was mainly in an oxic-dysoxic environment, which played an important role in the preservation of organic matter.

#### 5.2.3. Hydrothermal activity.

Sediments can record traces of hydrothermal activity, and the chemical characteristics sediments varies from region to region. For example, the enrichment of iron (Fe) and manganese (Mn) is often associated with hydrothermal fluids and forms iron-manganese sediments; whereas aluminum (Al) and titanium (Ti) oxides mostly indicate terrigenous clastic composition [77,78]. The (Cu + Co + Ni)×10-Fe-Mn and Zn-Ni-Co discrimination diagrams and the Al/ (Al + Fe + Mn) ratio are commonly used to reflect the influence of hydrothermal activity [79–81]. It is generally believed that the Al/ (Al + Fe + Mn) ratio usually less than 0.4 indicates that the hydrothermalism participated in diagenesis [79,82].

According to the discrimination criteria proposed by Adachi et al. (1986) [79] and Chen et al.(2004) [80], the “atypical hydrothermal activity” defined in this study is characterized by the higher Al/(Al + Fe + Mn) ratio than that of typical hydrothermal deposition (<0.4); the deviation of the Fe-Mn-Cu-Co-Ni elements from the typical hydrothermal region in the ternary diagram; the slight negative Eu anomaly in the distribution pattern of rare earth elements, different from the significant positive anomaly (δEu > 1.1) of typical hydrothermal fluids; and meanwhile accompanied by a great deal of terrigenous clastic input.

**Fig 10:**
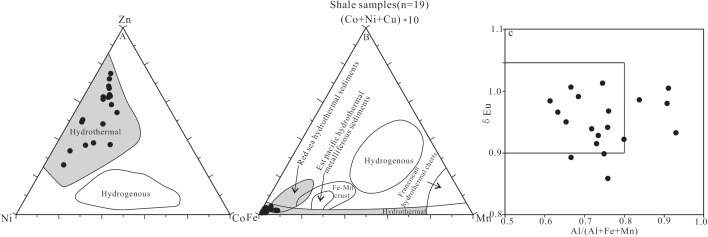
Dscrimination diagrams of hydrothermal activity influencing the shale in Member 1 of Keluke Formation. (A) Zn-Ni-Co; (B) (Cu + Co + Ni)×10-Fe-Mn; (C) δEu and Al/(Al + Fe + Mn).

**Fig 11:**
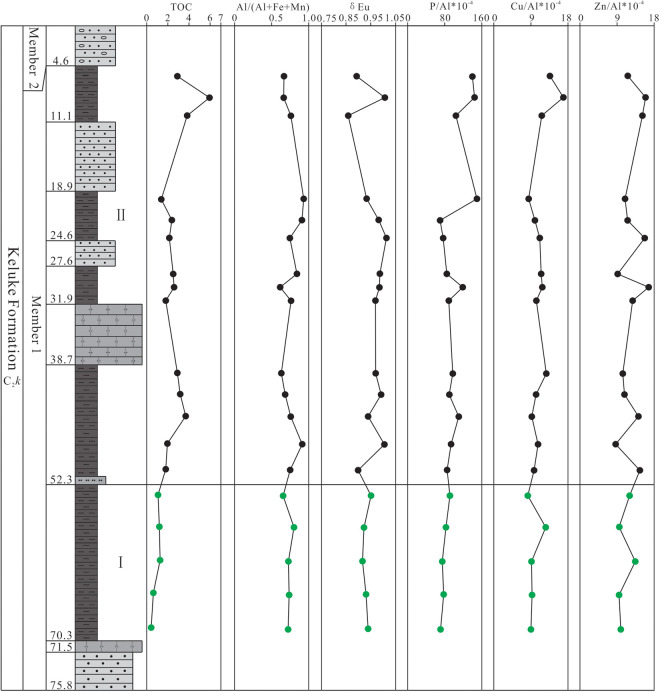
Vertical change trend of TOC, hydrothermal activity indicators and primary productivity indexes of shale samples from Member 1 of Keluke Formation.

In Fig 10A, B all the shale samples from Member 1 in the study area fall into the hydrothermal region. However, the Al/ (Al + Fe + Mn) ratios range from 0.61 to 0.93 (avg. 0.75; S2 Table, Fig 11), slightly greater than the threshold value for typical hydrothermal sediments (0.4), leading to the results inconsistent with those from the Fig 10A, B. This may be caused by the large amounts of terrigenous clastic input. The increase of the terrigenous clastic input amount can lead to the increase in Al_2_O_3_ content, and thus the increase of the Al/ (Al + Fe + Mn) ratio. The shale samples have higher contents of clay minerals (accounting for 32.00–53.50%; S1 Table) and Al_2_O_3_ (accounting for 13.59–21.44%, S2 Table), supporting the fact that the increase of terrigenous clastic input can cause the slightly greater Al/ (Al + Fe + Mn) ratio values (0.61–0.93). Therefore, we believe that the shale in Member 1 in the Huaitoutala area is affected by atypical hydrothermal activities.

Although the ratio of Al/(Al + Fe + Mn) may be relatively high due to the terrigenous clastic input, the intermittent input of hydrothermal fluids may still affect the sedimentary environment. The trace metals such as Fe and Mn released by the hydrothermal fluids may act as nutrients and stimulate the primary productivity of surface water body [83]. Meanwhile, the chemical composition changes of sediment pore water caused by the hydrothermal activity may promote the adsorption and preservation of organic matter [84]).

Eu anomaly can act as a geochemical indicator in evaluating hydrothermal activity and redox environment [73,85,86]. The positive Eu anomaly (δEu > 1) may be the result of the hydrothermal activity or the reduction condition, whereas the negative Eu anomaly (δEu < 1) may be related to the influence of the felsic igneous rocks or the oxidation condition [86–90]. However, in case δEu is siginificantly correlated with δCe, the Eu anomaly may be caused by diagenesis [91]. In this study, No correlation is found between δEu and δCe values (R = −0.019, n = 19; S4 Table) in the dark shale samples from Member 1 in the study area, indicating that the diagenesis influence on Eu anomaly (δEu) is limited or can be negligible. The δEu values of the shale samples from Member 1 range from 0.86 to 1.01 (avg. 0.95; S3 Table, Fig 11), almost all less than 1.0, showing a slightly negative Eu anomaly, indicating that no obvious hydrothermal activity influence during the formation process of the shale. Meanwhile, as discussed above, the Al/ (Al + Fe + Mn) values of the shale are greater than 0.4. Thus, the δEu and the Al/ (Al + Fe + Mn) values mutually verify that the dark shale in Member 1 are affected by atypical hydrothermal fluids, which might be related to the relatively great amount of terrigenous clastic input.

In order to effectively distinguish the relative contributions of the hydrothermal activity and the terrigenous input to the sedimentary system, this study constructed the δEu and Al/(Al + Fe + Mn) cross plot (Fig 10C). It can be seen that although the Al/(Al + Fe + Mn) ratios are generally high (0.61–0.93), 76% of the samples are concentrated in the transitional region with 0.5 < Al/(Al + Fe + Mn) ratio <0.8, 0.9 < δEu < 1.05, which shows the characteristics of the superimposition of hydrothermal fluid and terrigenous input. The samples in the transitional regions still show a hydrothermal tendency in the Fe-Mn-(Cu + Co + Ni)×10 diagram, indicating the presence of the original hydrothermal components. The great Al/(Al + Fe + Mn) ratio reflects that the terrigenous input. (with clay minerals accounting for 32%−53.5%) has diluted the hydrothermal components, but did not completely cover up their geochemical imprints.

### 5.3. Primary productivity

The primary productivity refers to the process in which organisms produce and accumulate organic matter from the external environment through life activity, which can affect the content of the organic matter in sediments and thus affect the enrichment of shale gas [92]. The content of the nutrient elements (such as P, Cu and Zn) in sedimentary water is an effective indicator of the primary productivity level [74]. Element P is an important nutrient element in the euphotic layer of seawater, cannot exist in the atmosphere as a gas phase [93], and can only be input through terrestrial rivers and winds [94]. The abundance of P in the crust is very low, but it can reach a high value in marine sediments, because P can accumulate in organism body through biological processe and then deposit to the seafloor. Therefore, P is widely used as an indicator of paleo-productivity. Depending on the redox condition of sedimentary water, P can be remained in an organic combination form, or diffuses upward from the sediments and returns to the water under sulfate reduction condition, or can be adsorbed on the surface of Fe oxide through the redox cycle of Fe, and then precipitates as authigenic phosphate minerals, which can affect the enrichment of P [95]. Almost all the studied samples are from the shale deposited in oxic-dysoxic environment, indicating a high P retention rate in sediments [74,95]. However, only the non-clastic derived P occurred in the sediments can represent the actural productivity. The P/Al ratio can effectively eliminate the influence of terrigenous clastic input, because Al usually comes from terrigenous clastic sources [56,96–98]. Therefore, the P/Al ratio is a reliable indicator of the primary productivity level.

The concentration of the nutrient trace mental elements (Cu and Zn) is low in seawater, but these elements play a key role in the photosynthesis process of marine phytoplankton [99]. These micro-nutrient elements are both the essential components in the physiological process of photosynthesis and the enzyme catalytic cofactors, which can affect the growth of phytoplankton [100,101]. The characteristics of Cu and Zn concentration change with water depth in the present seawater is similar to the vertical distribution feature of marine nutrient levels. That is, the concentration in the shallow water is low, while the concentration in the minimum oxygen-containing zone and in the bottom water is high [102,103]. This is because the growth of phytoplankton in the shallow water absorbs nutrients including Cu and Zn from the water, and the re-mineralization process of the organic matter in the deep water, especially in the seafloor sediments, can release these elements into the water. Therefore, the change in Cu and Zn concentration is considered to be closely related to the level of primary productivity of the water surface organisms [83]. The Cu/Al and Zn/Al ratios can eliminate the influence of the terrigenous clastic input and effectively reflect the level of the primary productivity.

**Fig 12:**
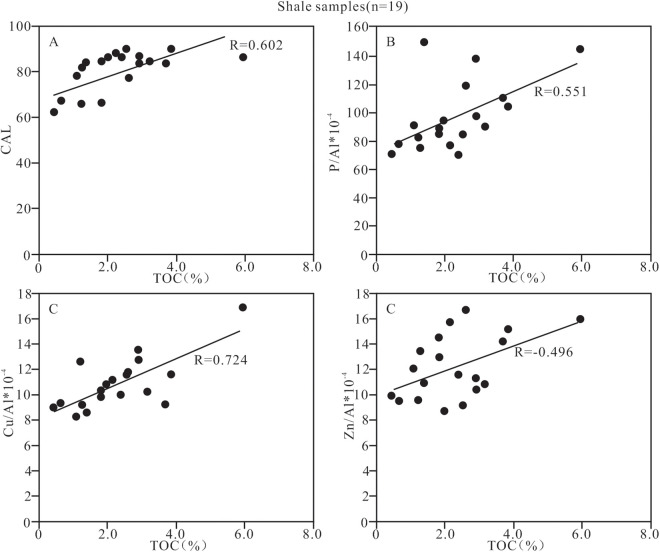
Relationship between TOC content and the paleo-climate and primary productivity indicators of shale samples from Member 1 of Keluke Formation.

The P/Al × 10^−4^ ratios of the shale samples from Member 1 in the study area range from 70.15 to 149.83 (avg. 97.49) (S2 Table, Fig 12), greater than the PAAS values (69.8) [42], indicating that the primary productivity of Member 1 is relatively high in the study area. The Cu/Al × 10^−4^ and the Zn/A × 10^−4^ ratios range from 8.26 to 16.93 (avg. 10.89) and 8.72 to 16.74 (avg. 12.27), respectively (S2 Table, Fig 12), also greater than the PAAS values (Cu/Al × 10^−4^ = 5.00 and Zn/A × 10^−4^ = 8.49) [42], again indicating that Member 1 had a high primary productivity during sedimentation period. In addition, the level of primary productivity in Sub-section II is greater than that of Sub-section I, showing an overall upward gradually increasing trend.

### 5.4. Factors controlling organic matter enrichment

In this study, the TOC content values of the shale in Member 1 vary widely, ranging from 0.43% to 5.96% (avg. 2.38%, n = 19; S1 Table), possibly representing the enrichment of different types of organic matter. But the organic matter enrichment mechanism in the shale is still unclear. The essence of organic enrichment is that the burial rate of organic matter is higher than the oxidation or degradation rate [16]. There are 2 main factors controlling the organic matter enrichment in sediments, namely the primary productivity of surface water and the redox condition of benthic water [24,31,33]. Besides, other factors, such as paleo-climate, paleo-water salinity, clastic input, sedimentation rate, sea level change, and hydrothermal activity may also affect the organic matter enrichment process, and have been widely concerned [25,27–30,32]. It is of great significance for the marine and terrestrial shale gas exploration in the study area to analyze the influence factors of organic matter enrichment, discuss the organic matter enrichment mechanism and establish the organic matter enrichment model.

#### 5.4.1. Control of paleoclimate and productivity on organic matter.

The suitable climate is a necessary condition for the growth of organisms. The unsuitable climate will lead to biological depletion and even biological extinction, but the prosperity of organisms plays a key role in the production of organic matter. The TOC content of Member 1 in the study area is positively correlated with CIA (R = 0.602, n = 19; Fig 12A, S4 Table), indicating that the climate plays an important role in the enrichment of organic matter. The warm and humid paleoclimate is conducive to the growth and prosperity of the marine organisms, and the remains of dead organisms can settle in the seabed and are eventually buried by sediments, resulting in the enrichment of large amounts of organic matter in the shale. The vertical distribution characteristics of the TOC and CIA values (Fig 3) also show that the high productivity corresponds to a warm and humid climate, which is conducive to the production and flourishing of organisms; the low productivity corresponds to a cold and dry climate, which is not conducive to the survival of organisms. The TOC is positively correlated with the indexes of P/Al, Cu/Al and Zn/Al (R = 0.551, 0.724 and 0.496, respectively, n = 19; Fig 12B–D and S4 Table), indicating that the higher the level of the primary productivity on the surface layer of the sedimentary water is, the more organic carbons can be fixed through photosynthesis and other ways, and correspondingly the higher the organic matter contents retain in the sediments. In summary, the enrichment of organic matter in the shale of Member 1 in this study is closely related to the paleo-climate and the primary productivity.

The P/Al and Corg/P ratios in samples from Member 1 of the Keluke Formation are significantly positively correlated with the TOC content, indicating that the enrichment of organic matter is jointly controlled by productivity and preservation conditions. By establishing a binary linear regression model: TOC = 0.019×(P/Al)+0.021×(Corg/P)+0.55(R2 = 0.68), the contributions of the primary productivity (P/Al) (30.4%), the preservation condition (Corg/P) (40.6%), and the synergy (29.0%) are calculated. These quantitative results indicate that the improvement of productivity and the enhancement of the dysoxic environment can jointly promote the enrichment of organic matter during the warm and humid period (Sub-section II), consistent with the “productivity-preservation synergy model” proposed by Algeo and Ingall (2007) [74].

In the marine-terrestrial transitional environment, the vigorous growth of siliceous organisms (such as radiolarians and diatoms) may constitute an important nutritional supplementation mechanism. The SiO_2_ content in the shale of Member 1 is high (41.41%−64.06%, avg. 58.68%), and the SiO_2_/Al_2_O_3_ ratio (2.04–4.68) is significantly higher than that in the typical continental clastic deposit (<2.0), suggesting the presence of biosilicon contribution. Modern analogical studies have shown that the vigorous growth of radiolarians can increase the primary productivity of water body by 30%−50% [30], and the sedimentation of their residues can promote the burial of organic matter. This siliceous organism driven “biological pump” effect, together withterrestrial nutrient input, constitutes the dual productivity mechanism in the marine-terrestrial transitional environment.

#### 5.4.2. Control of redox environment, paleosalinity and hydrothermal activity on organic matter.

**Fig 13:**
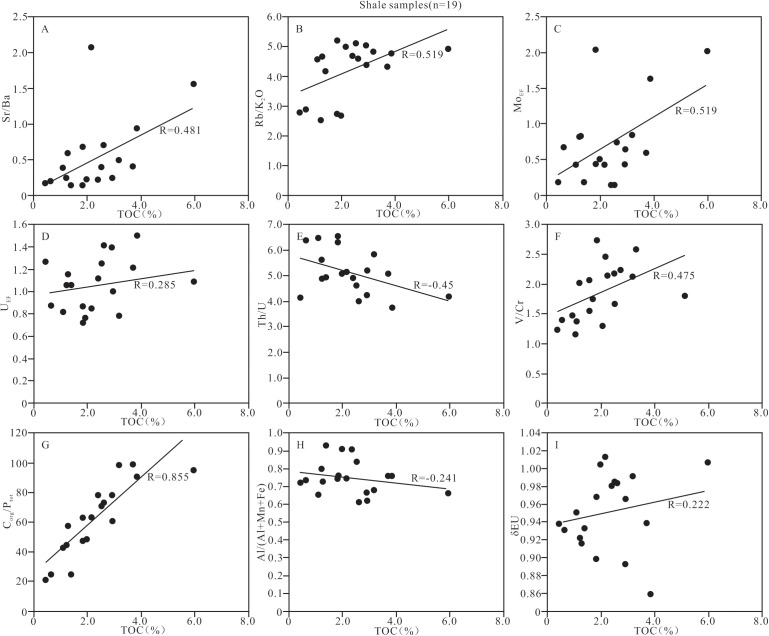
Identification plots showing the relationship between TOC content and the paleo-salinity indicators, redox condition indexes, and hydrothermal activity indexes of shale samples from Member 1 of Keluke Formation.

The redox environment plays a key role in the preservation of organic matter,with the anoxic environment being conducive to the preservation of organic matter. In addition, the high salinity environment is not conducive to the decomposition of organic matter, playing a certain role in the preservation of organic matter [20, 31−33, 69,}. In this study, the redox index EF_Mo_, V/Cr or C_org_/P ratio is positively correlated with the TOC content (R = 0.519, 0.475 and 0.855, respectively, n = 19; Fig 13C, F, G and S4 Table), but the EF_U_ and the Th/U ratio show no significant correlation with the TOC content (R = 0.285 and −0.450, respectively, n = 19; Fig 13D, E and S4 Table). These perplexing correlations reflect the particularity of the marine- terrestrial transitional environment. The vertical stratification effect of water body leads to a higher oxygen content in the surface water due to wave action, while the dysoxic environment is formed in the bottom water due to the degradation of organic matter. This vertical differentiation causes different indicators can capture the redox signals at different depths [69]. In addition, the relatively high clay mineral content in the shale samples indicates a faster sedimentation rate, which may lead to insufficient residence time of U at the oxidation-reduction interface and weaken the signal strength of U_EF_ [104]. Therefore, the organic matter enrichment of the Member 1 shale in the Keluke Formation is mainly controlled by the dysoxic conditions of the bottom water.

The Rb/K_2_O ratios range from 2.54 to 5.21, and the Sr/Ba ratios are 0.15–2.08, indicating that the shale of Member 1 was mainly formed in brackish water -salt water environment. Furthermore, the paleo-salinity indicators (Sr/Ba, Rb/K_2_O) are significantly positively correlated with TOC (R = 0.481 and 0.519, respectively, n = 19; Fig 13A, B and S4 Table), also indicating that the brackish water – salt water environment is obviously correlated with the enrichment of organic matter. In summary, the redox environment of water body during the shale sedimentation in Member 1 has little relationship with the enrichment of organic matter, while the change of paleosalinity is related to the enrichment of organic matter. In other words, high salinity water body is conducive to the organic matter enrichment, because it can enhance the preservation of organic matter. Modern high salinity water body is conducive to the preservation of organisms, which verifys that high salinity is beneficial to the preservation of organic matter.

Additionally, the geochemical identification plots (Fig 10A, B), along with the Al/(Al + Fe + Mn) ratio and δEu values, indicate that the shale from Member 1 may have been influenced by hydrothermal fluids, but the TOC content in the shale shows no significant correlation with the hydrothermal activity indexes of Al/ (Al + Fe + Mn) and δEu (R = −0.241 and 0.222, respectively, n = 19; Fig 13H, I and S4 Table). Therefore, the hydrothermal activity only has relatively limited contribution to the enrichment of organic matter.

#### 5.4.3. Control of terrigenous clastic input on organic matter.

**Fig 14:**
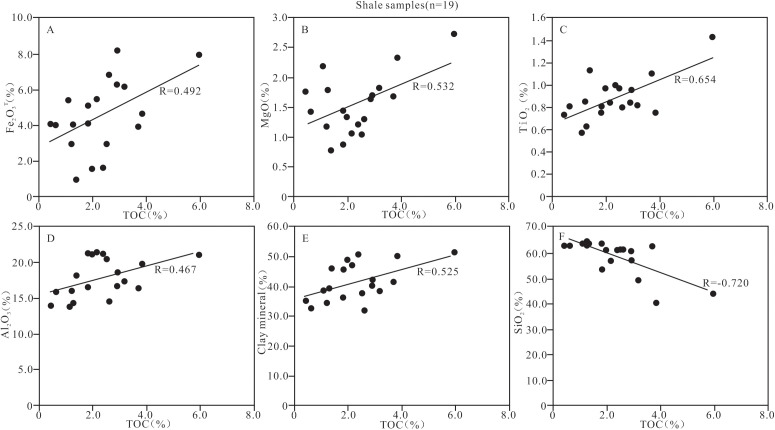
Identification plots showing the relationship between TOC and the clastic input indicators of shale samples from Member 1 of Keluke Formation.

**Fig 15:**
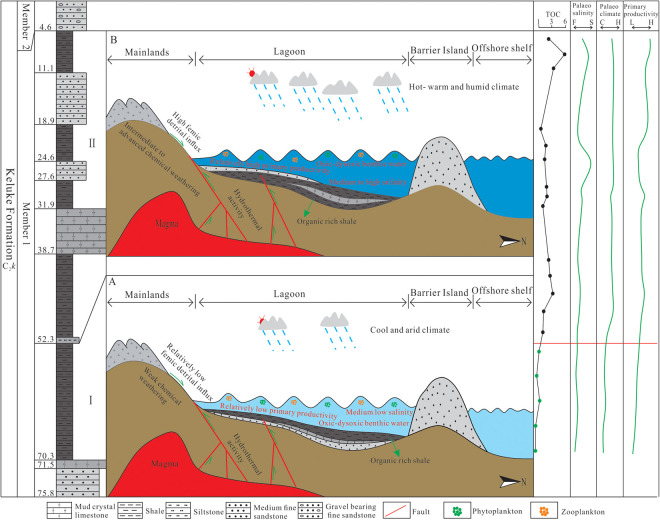
Schematic diagram of organic matter enrichment model of shale in Member 1 of Keluke Formation in the study area.

The clastic input has a dual effect on the enrichment of organic matter. The felsic clasitc input may dilute the organic matter [105], whereas the mafic clastic input may promote the survival and growth of the organisms [106], or enhance the input of clay minerals to adsorb organic matter [41,84]. The abundances of Fe_2_O_3_, MgO and TiO_2_ in the shale of Member 1 in the study area are high, and the ratios of Al_2_O_3_/TiO_2_ are low, indicating that the clastics in the shale are mainly from the basic source. The content of Fe_2_O_3_T, MgO or TiO_2_ is significantly positively correlated with the TOC content in the shale (R = 0.492, 0.532 and 0.654, respectively, n = 19; Fig 14A–C and S4 Table), indicating that the input of the mafic clastics promoted the enrichment of organic matter. In addition, the clay minerals and Al_2_O_3_ also show a positive correlation with the TOC content (R = 0.467 and 0.525, respectively, n = 19; Fig 15D, E, and S4 Table), indicating that the clay may provide sufficient surface area to adsorb organic matter and increase organic matter content. In contrast, the SiO_2_ and TOC content are significantly negatively correlated (R = −0.720, n = 19; Fig 14F and S4 Table), indicating that the input of felsic clastics has a dilutive effect on organic matter and is not conducive to organic matter enrichment.

#### 5.4.4 Influence of tectonic evolution on organic matter enrichment.

The eastern region of the NMQB was in the back-arc rift tectonic environment during the Carboniferous period [36,39]. Such type of structural background plays an important controlling role in the organic matter enrichment in the shale of Member 1 of the Keluke Formation, mainly due to the two aspects including the supply of nutrient elements and the favorable organic matter preservation conditions.

In the aspect of nutrient element supply, the upwelling of hydrothermal fluids caused by the back-arc rift activity released the nutrient elements such as Fe, Mn, and P into the sedimentary water body [18,30]. The slightly higher Al/(Al + Fe + Mn) ratio and the slight negative Eu anomaly in the study area indicate that atypical hydrothermal activity under the rift tectonic background has a certain modification effect on the sedimentary environment. Meanwhile, the tectonic subsidence caused by the rifting led to frequent marine intrusion events, promoting the mixed input of the terrestrial clastic materials and the marine nutrient elements. In addition, the chemical weathering index (CIA) of the shale samples studied is significantly positively correlated with the TOC content (R = 0.602, n = 19;) (Fig 12A and S2 Table), indicating that under warm and humid climatic conditions, the intense chemical weathering has significantly increased the supply flux of the terrestrial nutrients, which provides the material basis necessary for the prosperity of aquatic organisms.

In the aspect of organic matter preservation condition, the semi-enclosed marin basin environment formed by the back-arc rifting led to a significant stratification of water salinity, and this effect can effectively inhibit the benthic oxidation and provide favorable conditions for the preservation of the organic matter [69]. Furthermore, the rapid subsidence of the rift basin has significantly increased the sedimentation rate [16] and shortened the exposure time of organic matter in the oxidizing environment. The significant positive correlation between the high TOC content and the clay mineral content in Sub-section II of the Keluke Formation further verified that under the rapid deposition background, clay minerals can effectively promote the preservation of organic matter through adsorption [84].

### 5.5. Sedimentary model of Keluke Formation Shale

The systematic studies on the shale in the marine-terrestrial transitional Member 1 of the Upper Carboniferous Keluke Formation on the eastern NMQB, combined with the analysis of multiple indicators such as paleoclimate, paleo-salinity, redox condition, terrestrial clastic input, hydrothermal activity and primary productivity, have revealed the main controlling factors and evolution process of organic matter enrichment. The studies show that the enrichment of organic matter is controlled by the synergistic effect of paleoclimate, productivity, and preservation condition, among which the high primary productivity, the brackish – salt water environment and the dysoxic preservation condition in the warm and humid climate are the main factors controlling the organic matter enrichment. In addition, the terrestrial clastic input (clay mineral adsorption) also has an important influence on the enrichment of organic matter. Based on the vertical evolution characteristics, a two-section organic matter enrichment model of the Member 1 shale in the Keluke Formation is established (Fig 15).

Sub-section I is dominated by cold and arid climate, with less input of the terrigenous clastics, and thus less input of the nutrient elements into water, resulting in a relatively low productivity of the water body. Although hydrothermal process can import some nutrient elements into water, the organic matter content is still relatively low. Besides, the water body is dominated by a oxic-dysoxic environment with fresh-brackish water, making this sub-section not conducive to forming shale with high organic matter content (Fig 15A).

Sub-section II is dominated by warm, hot and humid climate, with enhanced terrigenous weathering, and large amounts of nutrient elements being imported into water. Additionally, hydrothermal process also inputs nutrient elements into water, resulting in rich nutrient elements in the water body, promoting the prosperity of organisms and improving the primary productivity, which leads to the increase of the organic matter content in sediments. Meanwhile, the high salinity, dysoxic and stratified water environment promote the preservation of organic matter, resulting in the formation of shale interval with highly anomalous, enriched organic matter. Compared with Sub-section I, Sub-section II has significantly higher organic matter content, and has been believed as the favorable interval for shale gas exploration in the study area (Fig 15B).

The two-section organic matter enrichment model of the Member 1 shale in the Keluke Formation exhibits distinctiveness and universality compared to the Marcellus Shale in the Appalachian Basin. The Kuke Formation’s two-section enrichment model is characterized by hydrothermal activity (Fe/Mn enrichment) and intense chemical weathering under a back-arc rift setting, distinguishing it from the biosiliceous-dominated mode typical of marine shales [107,108]. Simultaneously, it shares the stratigraphic regularity controlled by transgressive-regressive cycles with global marine-continental transitional systems.

## 6. Conclusion

(1) The chemical index of alteration (CIA) characteristics of Member 1 in the study area shows that the degree of the chemical weathering has experienced a variation trend from low to high. Of which, the terrigenous chemical weathering degree of Sub-section I is low, whereas the terrigenous chemical weathering degree of Sub-section II is moderate to strong. These indicate that the sedimentary period of Member 1 was dominated by the change of cold and arid to warm and humid climate, in which the climate of Sub-section I is colder and drier than that of Sub-section II.
(2) Studies on paleosalinity, redox index, hydrothermal activity diagram, terrigenous clastic input and primary productivity of Member 1 indicate that this Member is mainly in the oxic-dysoxic environment with brackish water – salt water. The terrigenous clastic input degree and primary productivity level show a variation trend from low to high, consistent with the paleoclimate evolution.
(3) The TOC content of the shale in Member 1 is siginificantly correlated with paleo-climate, paleo-salinity, primary productivity and clastic input, indicating that the enrichment of the organic matter in the shale is mainly controlled by paleo-climate, paleo-salinity, primary productivity and clastic input. Based on these findings, Member 1 is divided into 2 sub-sections – Sub-section I and Sub-section II, and a two-section organic matter enrichment model is established. Of which, Sub-section I was formed in a arid and cold climate, with low salinity oxic-dysoxic water, low primary productivity and low terrigenous clasitic input, and is an interval with low organic matter content; Sub-section II was formed in a hot, warm and humid climate, with high salinity and dysoxic water, high primary productivity and high terrigenous clasitic input, and contains high content of organic matter, and is an organic matter enriched interval.

## Supporting information

S1 Table TOC content and whole rock mineral compositions of dark shale samples from Member1 of Upper Carboniferous Keluke Formation in Huaitoutela area, eastern NMQB (%). (XLS)

S2 Table Content of major element (%) and ratio and parameter of representative elements of dark shale samples from Member1 of Upper Carboniferous Keluke Formation in Huaitoutela area, eastern NMQB. (XLS)

S3 Table Content of trace element (ppm) and ratio of representative elements of dark shale samples from Member1 of Upper Carboniferous Keluke Formation in Huaitoutela area, eastern NMQB. (XLS)

S4 Table Correlations of major, trace and rare earth elements and some related parameters of dark shale samples from Member1 of Upper Carboniferous Keluke Formation in Huaitoutela area, eastern NMQB. (XLS)

S5 Table EF value of trace elements of dark shale samples from Member1 of Upper Carboniferous Keluke Formation in Huaitoutela area, eastern NMQB. (XLS)
